# Meningeal solitary fibrous tumor cell states phenocopy cerebral vascular development and homeostasis

**DOI:** 10.21203/rs.3.rs-3164953/v1

**Published:** 2023-07-26

**Authors:** David Raleigh, Kanish Mirchia, Abrar Choudhury, Tara Joseph, Janeth Birrueta, Joanna Phillips, Aparna Bhaduri, Elizabeth Crouch, Arie Perry

**Affiliations:** University of California San Francisco; University of California San Francisco; University of California, San Francisco; University of California San Francisco; University of California San Francisco; UCSF; University of California Los Angeles; University of California San Francisco; UCSF

## Abstract

Meningeal solitary fibrous tumors (SFTs) are rare mesenchymal neoplasms that are associated with hematogenous metastasis, and the cell states and spatial transcriptomic architecture of SFTs are unknown. Here we use single-cell and spatial RNA sequencing to show SFTs are comprised of regionally distinct gene expression programs that resemble cerebral vascular development and homeostasis. Our results shed light on pathways underlying SFT biology in comparison to other central nervous system tumors and provide a framework for integrating single-cell and spatial transcriptomic data from human cancers and normal tissues.

The meningeal lining of the central nervous system gives rise to SFTs, which are rare, poorly understood cancers that harbor *NAB2-STAT6* gene fusions, and meningiomas, which are the most common primary intracranial tumors^1,2^. In contrast to meningiomas and other central nervous system (CNS) tumors, distant hematogenous metastasis are common for meningeal SFTs^3,4^. To elucidate cell states underlying the unique clinical behavior of meningeal SFTs in comparison to other CNS tumors, single-cell RNA sequencing was performed on 40,022 cells from 4 human SFT samples (Fig. 1a, Extended Data 1a-e, and Supplementary Table 1). Datasets were integrated and corrected for batch effects using Harmony^5^, and uniform manifold approximation and projection (UMAP) revealed a total of 12 cell clusters that were defined using automated cell type classification^6^, cell signature gene sets^7^, cell cycle analysis (Extended Data Fig. 1c), and differentially expressed cluster marker genes (Fig. 1b, Extended Data Fig. 1d, e, and Supplementary Table 1). Reduced dimensionality clusters of SFT microenvironment cells included macrophages (C6), immature neurons (C8), T cells (C9), and endothelia (C11) (Fig. 1a, b). SFT cell states were identified by expression of *NAB2* and *STAT6* (Extended Data Fig. 1d), and distinguished by differential expression of neuronal cell adhesion (*NCAM2*) or vascular (*VCAM1*) cell adhesion genes (C0, C1), cell stress genes (*EGR1*, *HSPA1A*) (C1), extracellular matrix (ECM) remodeling genes (*ADAMTS6*, *PLCG2*) (C3, C5), and protein synthesis genes (*RPS27A*, *RPL37*) (C4, C10), or by differential expression of endothelial genes (*CD34*) (C0, C1, C3, C5) and mural cell genes (*NOTCH3*, *PDGFRB*) (C7) (Fig. 1a, b). Single-cell trajectory analysis using RNA velocity^8,9^ suggested plasticity between SFT cell states (Fig. 1c), and pseudotemporal ordering of single-cells using Monocle^10^ supported the hypothesis that SFT cell fate decisions were dynamic and interchangeable (Fig. 1d). Cell-cell communication analysis of single-cell RNA sequencing data using CellChat^11^ showed receptor-ligand interactions throughout the SFT microenvironment, particularly between SFT cells expressing *VCAM1* (C2) and endothelia (C11), or between SFT cells expressing *NCAM2* (C0) and immature neurons (C8) (Fig. 1e).

SFTs and meningiomas can be challenging to distinguish using magnetic resonance imaging (MRI), but hematoxylin and eosin (H&E) and immunohistochemical (IHC) staining for STAT6 and SSTR2A can reliably distinguish SFTs from meningiomas^12,13^ (Fig. 1f). To define cell states distinguishing SFTs from meningiomas, Harmony and UMAP were used to integrate single-cell RNA sequencing data from SFT cells with 30,934 cells from 6 meningioma samples^14^ (Fig. 1g, Extended Data 2a-c, and Supplementary Table 2). Cell clusters were defined using automated cell type classification, cell signature gene sets, cell cycle analysis, and differentially expressed cluster marker genes (Fig. 1h, Extended Data Fig. 2b, c). The distribution of cell states was analyzed across SFT and meningioma samples (Fig. 1i). SFT cell states were distinguished by expression of *NAB2, STAT6, EGR1, VCAM1, NCAM2*, and the endothelial cell marker *CD34* (Fig. 1j). Meningioma cell states were distinguished by expression of *SSTR2A, PDGFRB*, and *NOTCH3* (Fig. 1j). NOTCH3 drives meningioma tumorigenesis and is diffusely expressed in high-grade meningiomas that are resistant to radiation^14^. In contrast, immunofluorescence (IF) confocal microscopy showed NOTCH3 expression was restricted to a minority of cells in the perivascular niche in SFTs (Extended Data Fig. 3a, b). These data demonstrate that meningiomas and meningeal SFTs are distinguished by minimally overlapping tumor cell states despite their shared anatomic origin and imaging characteristics.

SFTs range from moderately to highly cellular neoplasms that are comprised of closely approximated, haphazardly arranged tumor cells with varying amounts of intervening collagenous stroma and numerous thin walled, ectatic, branching blood vessels^1^. To define the spatial transcriptomic architecture of meningeal SFTs, spatial RNA sequencing of 55μm regions from continuous tiled arrays across 6mm cores was performed on 8 SFT samples using an approach that integrates approximately 10 cells per capture area^15,16^. DNA methylation profiling of SFTs (n = 8) and principal component (PC) analysis compared to DNA methylation profiling of meningiomas (n = 213)^17^ was used to validate SFT samples that were analyzed by spatial RNA sequencing (Extended Data Fig. 4). Tumor classification using DKFZ v12b6^18^ showed the samples analyzed using spatial transcriptomics clustered in the SFT methylation class with confidence scores > 0.8 (n = 2) and > 0.99 (n = 6). Targeted next-generation DNA sequencing^19^ of SFTs (n = 3) revealed *NAB2-STAT6* gene fusions (n = 2) with breakpoints at exon 7 (*NAB2*) and exon 18 (*STAT6*), and multiple large-scale chromosome gains or losses^20,21^.

The spatial transcriptomic architecture of SFTs were analyzed across 2021 CNS World Health Organization (WHO) histological grades^1^ (Fig. 2a and Extended Data Fig. 5a), or in patient-matched pairs of primary/recurrent or intracranial/metastatic samples (Fig. 2b and Extended Data Fig. 5b). Spatial deconvolution of cell types from single-cell RNA sequencing (Fig. 1a) across WHO histological grades demonstrated regionally distinct SFT cell states and microenvironment cell types despite relatively uniform histological characteristics, including spatial heterogeneity in the distribution of macrophages (C6) or cell adhesion (C0, C2), ECM remodeling (C3), protein synthesis (C4), and mural (C7) SFT cells (Fig. 2a). Spatial *NAB2* and *STAT6* expression were relatively uniform across WHO histological grades, but cell stress (*EGR1*) and vascular (*CD34, VWF, ACTA2*) gene expression programs were heterogeneous (Extended Data Fig. 5a). Cell-cell communication analysis weighted for spatial transcriptome proximity across spatial RNA sequencing clusters showed VCAM1 interactions were heterogeneous and correlated with spatial expression of Integrin ligands that bind to VCAM1 (Fig. 2a). SFT cell states and microenvironment cell types also demonstrated regionally distinct expression in patient-matched pairs of primary/recurrent or intracranial/metastatic samples, which showed temporal or spatial evolution in protein synthesis (C4) and ECM remodeling (C5) SFT cells, or ECM remodeling SFT cells (C3) and macrophages (C6) (Fig. 2b). Spatial *NAB2* and *STAT6* expression were uniform across primary/recurrent and intracranial/metastatic samples, but cell stress (*EGR1*) and vascular (*CD34, VWF, ACTA2*) gene expression programs were heterogeneous (Extended Data Fig. 5b). Thus, meningeal SFTs demonstrate regionally distinct intratumor heterogeneity in cell states, gene expression programs, and cell-cell interactions across WHO histological grades and paired primary/recurrent or intracranial/metastatic samples.

These single-cell RNA sequencing (Fig. 1a–e, Extended Data Fig. 1, and Supplementary Table 1) and spatial RNA sequencing data (Fig. 2a, b Extended Data Fig. 5a, b) suggest the cell states and spatial transcriptomic architecture of meningeal SFTs resemble vascular cell types. To test this hypothesis, cell types from single-cell RNA sequencing of perinatal human brain vasculature (139,134 cells from gestational weeks 15, 17, 18, 20, 22, or 23)^22^ or adult human brain vasculature (84,138 cells)^23^ were deconvolved from SFT single-cell or spatial transcriptomes. SFT spatial transcriptomes (n = 23,682) were integrated and corrected for batch effects using Harmony and UMAP, and spatial transcriptome clusters were defined using automated cell type classification, cell signature gene sets, cell cycle analysis, and differentially expressed cluster marker genes (Extended Data Fig. 6a-f and Supplementary Table 3). Deconvolution of cell types underlying perinatal cerebral vascular development or adult cerebral vascular homeostasis revealed homology between perinatal venous cells, perinatal capillary cells, perinatal fibroblasts, adult venous cells, and adult pericytes in SFT single-cell and spatial transcriptomes, with lower homology to other cerebral vascular cell types (Fig. 2c). Thus, the cellular architecture of SFTs phenocopies endothelial and mural cell types involved in cerebral vascular development and homeostasis.

In summary, these data shed light on pathways underlying SFT biology in comparison to other intracranial meningeal tumors and provide a framework for integrating single-cell and spatial transcriptomic data from human cancers and normal tissues. We show SFTs are comprised of plastic cell states, regionally distinct gene expression programs, and cell-cell interactions that resemble cerebral vascular development and homeostasis. These data provide new insights into a rare, poorly understood cancer with a high rate of hematogenous metastasis that is unique in comparison to meningiomas and other tumors of the central nervous system.

## Methods

This study complied with all relevant ethical regulations and was approved by the UCSF Institutional Review Board (13–12587, 17–22324, 17–23196, and 18–24633). As part of routine clinical practice, all patients who were included in this study signed a written waiver of informed consent to contribute de-identified tissue for research.

### Single-cell RNA sequencing and analysis

Single cells were isolated from human SFT or meningioma samples, as previously described^17^. Single-cell suspensions were processed for single-cell RNA sequencing using the Chromium Single Cell 3’ GEM, Library & Gel Bead Kit v3.1 (1000121, 10x Genomics) and a 10x Chromium or Chromium X controller, using the manufacturer recommended default protocol and settings for a target recovery of 5,000 cells per sample. Libraries were sequenced on an Illumina NovaSeq 6000, targeting >50,000 reads per cell, at the UCSF Center for Advanced Technology. Library demultiplexing, read alignment, identification of empty droplets, and UMI quantification were performed using CellRanger (https://github.com/10xGenomics/cellranger). Cells were filtered based on the number of unique genes, and single-cell UMI count data were preprocessed in R with the Seurat package (v4.3.0)^24,25^ using the sctransform workflow. Dimensionality reduction was performed using PC analysis. UMAP and Louvain clustering were performed on the reduced data, followed by marker identification and differential gene expression.

Clusters were defined using a combination of automated cell type classification^6^, cell signature gene sets^7^, cell cycle analysis, and differentially expressed cluster marker genes. The scType R package was used for automated cell type classification, with default parameters and an augmented list incorporating package-provided human ‘Brain’ marker genes specific to each cell type^6^. Gene set enrichment analysis was performed on clusters using cell type signature gene sets from from MSigDB (https://www.gsea-msigdb.org/gsea/msigdb) with the fgsea R package (Bioconductor v3.16). Cell cycle phases of individual cells were assigned with the ‘CellCycleScoring’ function in Seurat, using single-cell cell cycle marker genes^26^.

Meningeal SFT and meningioma single-cell samples were aligned to the GRCh38 human reference genome; filtered to cells with greater than 250 unique genes, less than 7,500 unique genes, and less than 25% of reads attributed to mitochondrial transcripts; scaled based on regression of UMI count and percentage of reads attributed to mitochondrial genes per cell; and corrected for batch effects using Harmony^5^. Parameters for downstream analysis were a minimum distance metric of 0.2 for UMAP, resolution of 0.15 for Louvain clustering, determined using clustree (v0.5.0, analyzing resolutions 5, 2, 1, 0.9, 0.8, 0.7, 0.6, 0.5, 0.4, 0.3, 0.25, 0.2, 0.15, 0.1, 0.0), with a minimum difference in fraction of detection of 0.25 and a minimum log-fold change of 0.25 for marker identification.

Deconvolution of SFT cell types from reference perinatal or adult vascular cell type single-cell RNA sequencing dataset was performed using SCDC (v0.0.0.9000)^27^. Single-cell transcriptomic data from the reference datasets were subsampled to 1000 cells per cluster, and the top differentially expressed genes were selected for each cell type. Using this expression set, SFT single cells were deconvolved to yield a matrix with predicted proportions of cell type for each cell, which were visualized using feature plots.

Gene enrichment analysis was performed using a list of 50 most differentially expressed candidate genes from previously published single-cell perinatal or adult vascular and mural cell types^22,23^. Average counts per cell were summarized, scored as mean, and visualized using feature plots.

Cell-cell communication networks were inferred and visualized using the CellChat R package (v1.5.0)^11^. Briefly, differentially expressed signaling genes were identified, noise was mitigated by calculating the ensemble average expression, intercellular communication probability was calculated by modeling ligand-receptor interactions using the law of mass action, and statistically significant communications were identified. The CellChat commands ‘computeCommunProb’, ‘computeCommunProbPathway’, and ‘aggregateNet’, were used for analysis, and ‘netVisual_aggregate’ was used for visualization.

Trajectory analyses was performed using monocle3 (v1.3.1)^10,28,29^ for pseudotime, and velocyto (v0.17.16)^8^ with scVelo (v0.2.5)^9^ for RNA velocity. For pseudotime analysis, data were normalized followed by UMAP dimensionality reduction as described above. The ‘cluster_cells’ and ‘learn_graph’ monocle commands were used with default parameters and cells were ordered along pseudotime after manually selecting a root node (based on cluster, cell type, and cell cycle information). For RNA velocity analysis, velocyto was used to generate loom files with spliced and unspliced mRNA count information. scVelo was used to filter and normalize gene expression using criteria “min_shared_counts=3’, and ‘n_top_genes=2000’ prior to computing RNA velocity and latent time. RNA velocity was visualized by projecting on to the UMAP generated using R and Seurat.

### Spatial RNA sequencing and analysis

Spatial transcriptomic profiling was performed on FFPE sections using the 10x Genomics Visium Spatial assay (1000336, v1). 6 mm cores were mounted within capture areas on Visium glass slides, deparaffinized, stained with H&E, and imaged at the Gladstone Institutes Histology Core. Libraries were prepared according to manufacturer instructions at the Gladstone Institutes Genomics Core. Libraries were sequenced on an Illumina NovaSeq 6000 instrument at the UCSF Center for Advanced Technology. Sequencing was performed with the recommended protocol (read 1: 28 cycles, i7 index read: 10 cycles, i5 index read: 10 cycles, read 2: 91 cycles). FASTQ sequencing files and histology images were processed using the 10x SpaceRanger pipeline and the Visium Human Transcriptome Probe Set v1.0 GRCh38–2020-A. Data were visualized using the 10x Loupe Browser software (v6.4.0) and Seurat package (v4.3.0)^24,25^.

**Figure 1:**
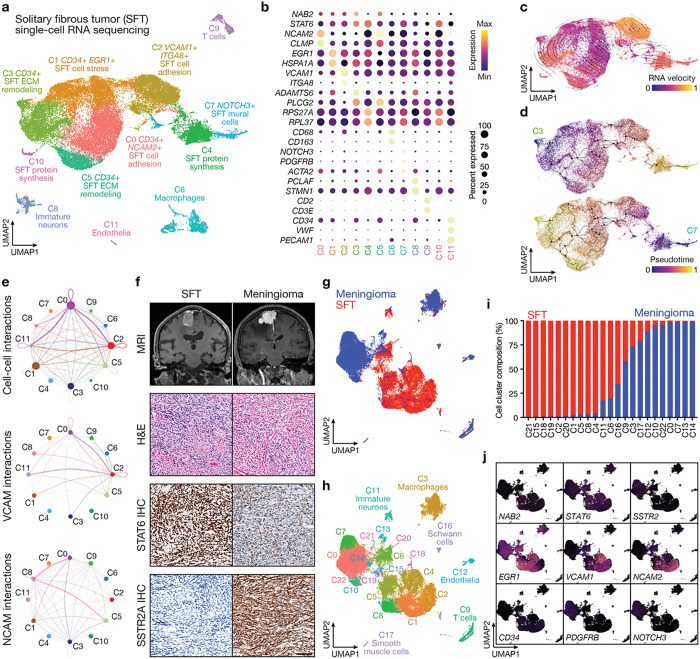
Meningeal solitary fibrous tumor cell states resemble vascular cells. **a**, Single-cell RNA sequencing UMAP of 40,022 transcriptomes from meningeal SFT samples showing tumor cell states and microenvironment cell types. ECM, extracellular matrix. **b**, Dot plot showing differentially expressed cell cluster marker genes across SFT cell states and microenvironment cell types. Cluster colors as in **a**. **c**, RNA velocity trajectory analysis of SFT cell states. **d**, Pseudotime trajectory analysis of SFT cell states using clusters at the termini of UMAP space (C3, C7) as the starting points in SFT cell state evolution. **e**, Inference of cell-cell interactions and VCAM or NCAM interactions in SFTs using single-cell RNA sequencing cell-cell communication analysis. **f**, MR imaging, H&E staining, and IHC staining for STAT6 or SSTR2A in SFT versus meningioma. Scale bar, 100μm. **g**, **h**, Single-cell RNA sequencing UMAPs of 70,956 transcriptomes from SFT and meningioma samples shaded by tumor type of origin in **g** or by cell cluster in **h**. **i**, Cell cluster distribution across SFT and meningioma samples analyzed using single-cell RNA sequencing. **j**, Feature plots showing differentially expressed genes across UMAP clusters from SFT and meningioma samples analyzed using single-cell RNA sequencing.

Spaceranger generated filtered feature matrices were imported into a Seurat object (v4.3.0, arguments min.cells=3, min.features=100) using R (v4.2.1) and RStudio (v2022.07.2 Build 576). The individual count matrices were normalized based on nFeature_RNA count (subset=nFeature_RNA>1500 and nFeature_RNA<9500) with less than 10% of reads attributed to mitochondrial transcripts and integration using Harmony (v0.1.1). Parameters for downstream analysis were a minimum distance metric of 0.2 for UMAP, resolution of 0.2 for Louvain clustering, determined using clustree (v0.5.0, analyzing resolutions 5, 2, 1, 0.9, 0.8, 0.7, 0.6, 0.5, 0.4, 0.3, 0.25, 0.2, 0.15, 0.1, 0.0), and a minimum difference in fraction of detection of 0.25 and a minimum log-fold change of 0.25 for marker identification. UMAP projections and cluster distributions were visualized in the Loupe browser as needed, after combining spatial transcriptomic data from individual capture areas using the 10x Spaceranger aggr pipeline (v2.0.0). Deconvolution of SFT cell types from reference SFT single-cell RNA sequencing was performed using SCDC (v0.0.0.9000)^27^. To do so, each spatial transcriptome was treated as a pseudobulked RNA sequencing dataset and leveraged against known cell types from reference single-cell RNA sequencing datasets comprised of 40,022 cells from 4 human SFT samples (Fig. 1a), perinatal human brain vasculature (139,134 cells from gestational weeks 15, 17, 18, 20, 22, or 23)^22^, or adult human brain vasculature (84,138 cells)^23^. Single-cell transcriptomic data were subsampled to 1000 cells per cluster, and the top differentially expressed genes were selected for each cell type. Using this expression set, spatial transcriptomes were deconvolved to yield a matrix with predicted proportions of cell type for each spatial transcriptome, which were visualized using spatial feature plots.

Gene enrichment analysis was performed using a list of 50 most differentially expressed candidate genes from previously published single-cell perinatal or adult vascular and mural cell types^22,23^. Average counts per spatial transcriptome were summarized, scored as a mean, and visualized using spatial feature plots.

The cell-cell communication network was inferred and visualized using the CellChat R package (v1.5.0)^11^ similar to the method used for single cell RNA sequencing samples. Briefly, differentially expressed signaling genes were identified, noise was mitigated by calculating the ensemble average expression, intercellular communication probability was calculated by modeling ligand-receptor interactions using the law of mass action, and statistically significant communications were identified. The CellChat commands ‘computeCommunProb’, ‘computeCommunProbPathway’, and ‘aggregateNet’ were used for analysis, and ‘netVisual_aggregate’ was used for visualization. ‘computeCommunProb’ was run using spatial information from the Visium assay, including spatial dot coordinates and scale.factors for the fiducials and low/high-resolution tissue images.

### DNA methylation profiling and analysis

Genomic DNA underwent bisulfite conversion using the EZ DNA Methylation kit (D5004, Zymo Research), followed by amplification, fragmentation, and hybridization to Infinium EPIC 850k Human DNA Methylation BeadChips (20020530, Illumina) according to manufacturer’s instructions at the University of Southern California Molecular Genomics Core. Bioinformatic analysis was performed in R (v4.2.1). SFT or meningioma DNA methylation data were preprocessed using the minifi pipeline^30^. In brief, probes were filtered and analyzed using normal-exponential out-of-band background correction, nonlinear dye bias correction, p-value with out-of-band array hybridization masking, and β value calculation (β=methylated/[methylated+unmethylated]). Principal component analysis was performed on the β methylation values from minfi pre-processing pipeline in R. Variable probes were identified from the first three principal components (PCs). PCs greater than 4 contributed to less than 5% of β value variance. The top 2000 probes were selected for analysis by ranking the absolute gene loading score values within PCs and the tumors were projected along the first two PCs.

### Targeted next-generation DNA sequencing and analysis

Targeted DNA sequencing was performed using the UCSF500 NGS panel, as previously described^19^. In brief, this capture-based next-generation DNA sequencing assay targets all coding exons of 479 cancer-related genes, select introns, and upstream regulatory regions of 47 genes to enable detection of structural variants such as gene fusions and DNA segments at regular intervals along each chromosome to enable genome-wide copy number and zygosity analyses, with a total sequencing footprint of 2.8 Mb. Multiplex library preparation was performed using the KAPA Hyper Prep Kit (07962355001, Roche). Hybrid capture of pooled libraries was performed using a custom oligonucleotide library (Nimblegen SeqCap EZ Choice). Captured libraries were sequenced as paired-end reads on an Illumina NovaSeq 6000 at >200x coverage for each sample. Sequence reads were mapped to the reference human genome build GRCh37 (hg19) using the Burrows-Wheeler aligner (v0.7.17). Recalibration and deduplication of reads was performed using the Genome Analysis Toolkit (v4.3.0.0). Coverage and sequencing statistics were determined using Picard (v2.27.5), CalculateHsMetrics, and CollectInsertSizeMetrics. Single nucleotide variant and small insertion/deletion mutation calling was performed with FreeBayes, Unified Genotyper, and Pindel. Large insertion/deletion and structural alteration calling was performed with Delly. Variant annotation was performed with Annovar. Single nucleotide variants, insertions/deletions, and structural variants were visualized and verified using Integrative Genome Viewer (v.2.16.0). Genome-wide copy number and zygosity analysis was performed by CNVkit and visualized using NxClinical (Biodiscovery, v6.0).

### Histology and microscopy

For adult human tissue samples, deparaffinization and rehydration of 5μm formalin-fixed, paraffin-embedded (FFPE) tissue sections and H&E staining were performed using standard procedures. Immunostaining was performed on an automated Ventana Discovery Ultra staining system and detection was performed with Multimer HRP (Ventana Medical Systems) followed by fluorescent detection (DISCOVERY Rhodamine and CY5) or DAB.

Immunostaining for NOTCH3 was performed using mouse monoclonal NOTCH3/N3ECD primary antibody (MABC594, Millipore Sigma, 1:25–1:100) with incubation for 32min following CC1 antigen retrieval for 32min. For dual staining, primary antibody incubations were carried out serially with inclusion of positive, negative, and single antibody controls. Following staining for NOTCH3/N3ECD, tissue sections were stained with primary antibodies recognizing SMA (ab7817, Abcam, mouse polyclonal, 1:30,000) for 32min or VWF (A0082, Dako, rabbit polyclonal, 1:1,000) for 20min. All IF experiments were imaged on a LSM 800 confocal laser scanning microscope with Airyscan (Zeiss) and analyzed using ImageJ.

Clinically validated immunohistochemistry for STAT6 (ab32520, Abcam, 1:100 dilution, YE361 clone), SSTR2A (ab134152, Abcam, 1:2000 dilution, UMB1 clone), were performed at UCSF on core mounts with appropriate controls using a Leica Bond III platform and imaged using light microscopy on an Olympus BX43 microscope with standard objectives. Images were obtained and analyzed using the Olympus cellSens Standard Imaging Software package (v1.16).

### Statistics

All experiments were performed with independent biological replicates and repeated, and statistics were derived from biological replicates. Biological replicates are indicated in each figure panel or figure legend. No statistical methods were used to predetermine sample sizes, but sample sizes in this study are similar or larger to those reported in previous publications. Data distribution was assumed to be normal, but this was not formally tested. Investigators were blinded to conditions during clinical data collection and analysis. Bioinformatic analyses were performed blind to clinical features, outcomes, or molecular characteristics. The clinical samples used in this study were retrospective and nonrandomized with no intervention, and all samples were interrogated equally. Thus, controlling for covariates among clinical samples was not relevant. Cells and animals were randomized to experimental conditions. No clinical, molecular, or cellular data points were excluded from the analyses.

### Reporting summary

Further information on research design is available in the Nature Research Reporting Summary linked to this article.

## Figures and Tables

**Figure 2:**
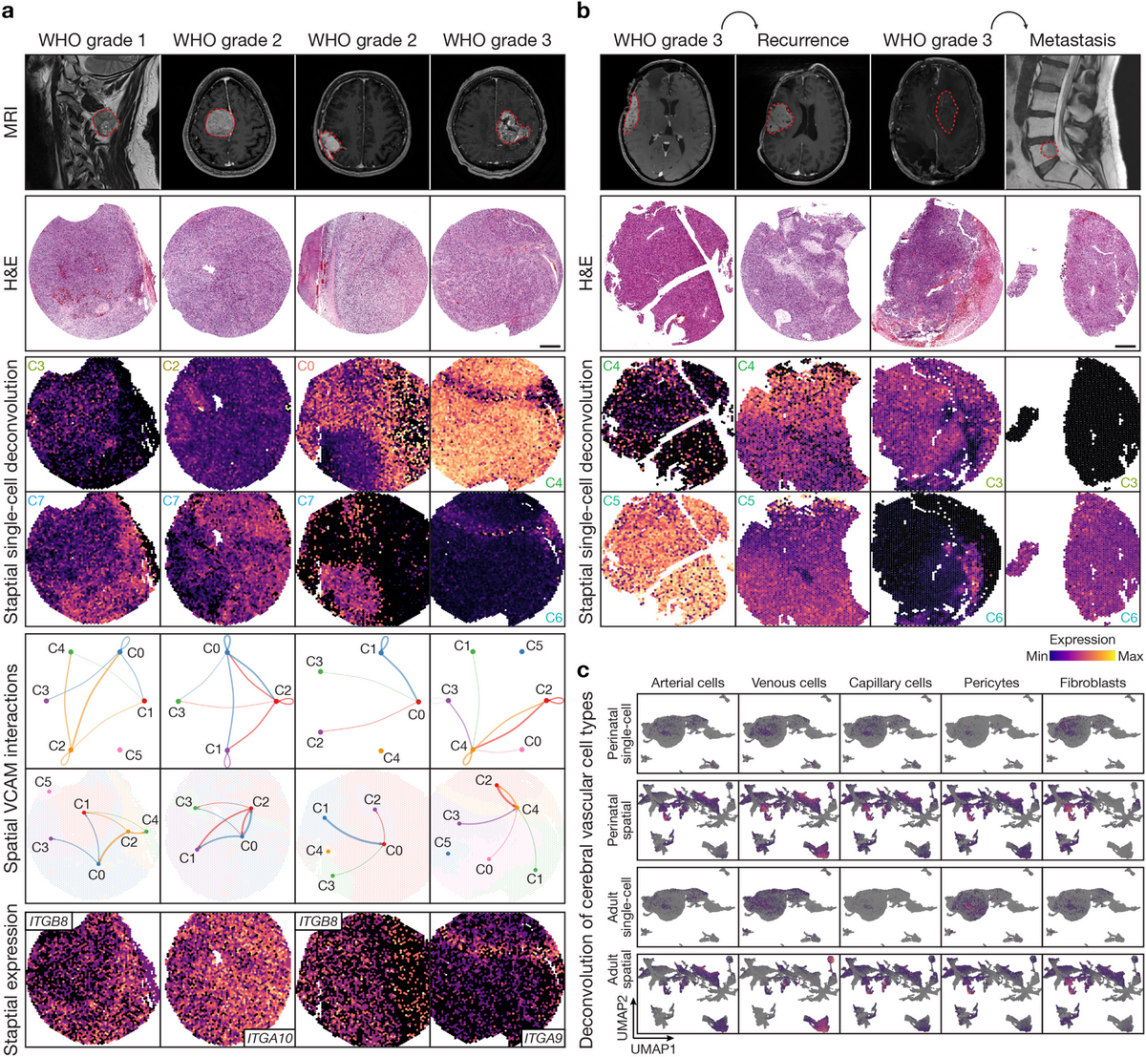
Meningeal solitary fibrous tumor cell states are regionally distinct and phenocopy cerebral vascular development or homeostasis. **a**, **b**, MR imaging, H&E staining, spatial deconvolution of cell types from single-cell RNA sequencing, VCAM signaling networks weighted for spatial transcriptome proximity across spatial RNA sequencing clusters, and spatial expression of Integrin VCAM ligands across SFT CNS WHO histological grades in **a**or across patient-matched pairs of primary/recurrent and intracranial/metastatic samples in **b**. Scale bars, 10μm (top) and 100μm (bottom). **c**, Feature plots showing deconvolved perinatal or adult cerebral vascular cell types across single-cell RNA sequencing or spatial RNA sequencing UMAP clusters from SFT samples. Single-cells or spatial transcriptomes without concordance to cerebral vascular cell types are shown in grey.

## Data Availability

SFT single-cell RNA sequencing (n=4), spatial RNA sequencing (n=8), and targeted next-generation DNA sequencing data (n=3) have been deposited to the Sequence Read Archive under BioProject ID PRJNA986661 (http://www.ncbi.nlm.nih.gov/bioproject/986661). SFT DNA methylation profiling data (n=8) have been deposited to the NCBI Gene Expression Omnibus under accession GSE235922 (https://www.ncbi.nlm.nih.gov/geo/query/acc.cgi?acc=GSE235922). Previously-generated meningioma RNA sequencing, single-cell RNA sequencing, and DNA methylation profiling data have been deposited to the NCBI Gene Expression Omnibus under accessions GSE183655 (https://www.ncbi.nlm.nih.gov/geo/query/acc.cgi?acc=GSE183655), GSE183656 (https://www.ncbi.nlm.nih.gov/geo/query/acc.cgi?acc=GSE183656), GSE212666 (https://www.ncbi.nlm.nih.gov/geo/query/acc.cgi?acc=GSE212666), GSE183653 (https://www.ncbi.nlm.nih.gov/geo/query/acc.cgi?acc=GSE183653), and GSE101638 (https://www.ncbi.nlm.nih.gov/geo/query/acc.cgi). The publicly available dataset GRCh38 was used in this study (hg38, https://www.ncbi.nlm.nih.gov/assembly/GCF_000001405.39/). Source data are provided with this study.
